# Development of a multi-laboratory integrated predictive model for silicosis utilizing machine learning: a retrospective case-control study

**DOI:** 10.3389/fpubh.2024.1450439

**Published:** 2025-01-15

**Authors:** Guo-kang Sun, Yun-hui Xiang, Lu Wang, Pin-pin Xiang, Zi-xin Wang, Jing Zhang, Ling Wu

**Affiliations:** ^1^Department of Laboratory, West China School of Public Health and West China Fourth Hospital, Sichuan University, Chengdu, China; ^2^Sichuan International Travel Health Care Center (Chengdu Customs Port Outpatient Department), Chengdu, China; ^3^Department of Laboratory, Akesu Center of Disease Control and Prevention, Akesu, China; ^4^Department of Laboratory, Xiping Community Healthcare Center of Longquanyi District, Chengdu, China; ^5^Department of Laboratory, Wangjiang Hospital, Sichuan University, Chengdu, China

**Keywords:** silicosis, early diagnostics, liquid biopsy, biomarkers, machine learning

## Abstract

**Objective:**

Due to the high global prevalence of silicosis and the ongoing challenges in its diagnosis, this pilot study aims to screen biomarkers from routine blood parameters and develop a multi-biomarker model for its early detection.

**Methods:**

A case-control study was conducted to screen biomarkers for the diagnosis of silicosis using LASSO regression, SVM and RF. A sample of 612 subjects (half cases and half controls) were randomly divided into training and test groups in a 2:1 ratio. Logistic regression analysis and receiver operating characteristic (ROC) curves were used to construct a multiple biomarker-based model for the diagnosis of silicosis, which was applied to both the training and the testing datasets.

**Results:**

The training cohort revealed significant statistical differences (*P* < 0.05) in multiple hematologic parameters between silicosis patients and healthy individuals. Based on machine learning, eight silicosis biomarkers were screened and identified from routine blood cell, biochemical and coagulation parameters. D-dimer (DD), Albumin/Globulin (A/G), lactate dehydrogenase (LDH) and white blood cells (WBC) were selected for constructing the logistic regression model for silicosis diagnostics. This model had a satisfactory performance in the training cohort with an area under the ROC curve (AUC) of 0.982, a diagnostic sensitivity of 95.4%, and a specificity of 92.2%. In addition, the model had a prediction accuracy of 0.936 with an AUC of 0.979 in the independent test cohort. Moreover, the diagnostic accuracies of the logistic model in silicosis stages 1, 2, and 3 were 88.0, 95.4, and 94.3% with an AUC of 0.968, 0.983, and 0.990 for silicosis, respectively.

**Conclusion:**

A diagnostic model based on DD, A/G, LDH and WBC is successfully proposed for silicosis diagnostics. It is cheap, sensitive, specific, and preliminarily offers a potential strategy for the large-scale screening of silicosis.

## Introduction

Silicosis is a chronic, debilitating and irreversible fibrotic lung disease resulting from the inhalation of crystalline silica particles, posing a significant occupational health challenge worldwide. Characterized by inflammation, nodular lesions, and pulmonary fibrosis, silicosis leads to severe complications such as tuberculosis, respiratory failure, and increased mortality (1). The mechanism of silica-induced pulmonary lesions is multifaceted, involving direct cytotoxic effects on macrophages, cytokine and chemokine production, cell apoptosis/necrosis, pulmonary fibrosis, lysosomal rupture, macrophage surface receptor activation, generation of reactive oxygen species and inflammasome activation (2–4). Despite all efforts to improve occupational health, silicosis is still one of the most prevalent occupational diseases in the world, afflicts workers in hazardous environments, and kills thousands of people every year (5). Even worse, silicosis cases still increase obviously in some specific industries, such as jewelry, glass production, nanomaterial production, and manufacturing (6, 7). Recent research indicated that over 230,000 workers in China were directly exposed to respirable crystalline silica (RCS). Silicosis has become a huge burden in China, which demonstrated the highest age-standardized rate (ASR) of silicosis (113.149, 95% UI: 92.924–136.700, per 100,000 population, in 2019) (8, 9). A recent study from Xin Liu et al. shows that ASRs due to silicosis vary widely among 204 countries worldwide. Of these, four countries (Italy, Chile, North Korea, and China) had an ASIR of more than 1/100,000, and 101 countries had an ASIR of < 0.1/100,000, with the lowest being Iceland at < 0.001/100,000, and the highest being China at 5.92/100,000. Similarly, Iceland had the lowest ASPR, while China still had the highest (113.15/100,000), followed by North Korea, Chile, Mexico, Italy, Brazil, Palau, Albania and Slovenia (10). Therefore, early diagnostics and therapeutics are critical for slowing disease progression and improving patient quality of life. Currently, the chest X-ray test is the gold standard of silicosis diagnosis. Additional tests, such as a breathing test, chest CT scan, bronchoscopy test, and tissue biopsy of the lungs, may be required to assess silicosis and associated diseases (11, 12). However, these traditional examinations rely heavily on pulmonary functional abnormalities and radiographic changes that occur in the moderate and late stages of silicosis disease. To reduce delays in diagnosis and treatment, liquid biopsy techniques based on various biomarkers are expected to reliably screen and detect the early stages of silicosis without invasive and expensive examinations (13).

Screening and identification of key biomarkers for specific diseases are the most crucial in liquid biopsy (14). In the past decade, various biomarkers have been proposed for the early diagnosis and management of silicosis (15). Tumor necrosis factor–α (TNF–α) and interleukins (ILs) are the first to be discovered as contributors in silicosis progression and potential biomarkers of silicosis diagnostics. After capturing silica particles, alveolar macrophages will release many inflammatory cytokines, such as TNF-α and ILs. These mediators may maintain the inflammatory process, and then damage cells and the extracellular matrix (16). The marked increases in TNF-α and ILs could be observed in silicosis patients before the onset of clinical signs and radiographic changes, making them promising biomarkers for early diagnosis of silicosis (17). In addition to inflammatory markers, some peripheral markers of epithelial destruction have also been regarded as potential biomarkers of silicosis (16). For example, Clara cell protein 16 (CC16) plays vital roles in anti-inflammatory, anti-fibrotic, and immunosuppressive processes. Serum CC16 level was reported to decrease in silicosis patients due to inflammation and cell damage (18, 19). Krebs von den Lungen-6 (KL-6) is a mucin-like glycoprotein on type 2 pneumocytes. The serum level of KL-6 can reflect the extent of alveolar epithelial damage. It is a potential biomarker for lung fibrosis in general, but its value for specific diagnosis of silicosis is not yet defined (20–22). Besides, mucin 5B (MUC5B), neopterin, and matrix metalloproteinases (MMPs) have also been considered as potential biomarkers of silicosis (23–25).

To date, several silicosis-specific biomarkers have been discovered in cohort studies and animal experiments. However, accurate detection of these biomarkers requires delicate instruments, expensive reagents, and professionally trained analysts, making large-scale screening difficult to implement in occupational populations. Since the combination of multiple biomarkers could improve the specificity and sensitivity of early diagnostics (14), this pilot study aims to construct a rapid and cost-effective multiple biomarker-based model for diagnosing silicosis based on routine laboratory results.

## Methods

### Study design

This retrospective case-control study aims to develop a multiple blood biomarker model for diagnosing silicosis in silica-exposed workers.

### Setting

The West China School of Public Health/West China Fourth Hospital of Sichuan University is one of the most famous public health colleges in China and the only tertiary Grade A occupational disease specialist hospital among the 44 commissioned hospitals of the National Health Commission. It features geriatric medicine, occupational disease, and minimally invasive treatment. The West China Fourth Hospital has diagnostic qualifications for pneumoconiosis and all ten major occupational diseases, which can meet the testing needs of all occupational disease projects in the industry.

### Participants

We extracted a random sample of patients hospitalized in the West China Fourth Hospital of Sichuan University between 2021 and 2024 who had documented exposure to silica dust in occupational settings. Since silicosis is caused by silica dust exposure and is usually diagnosed as an occupational disease, we controlled for these factors to reduce bias. Cases were defined as patients diagnosed with silicosis based on the revised edition (2011) of the International Labor Office guideline (26). Controls were defined as patients who were silicosis-free. We excluded all patients with severe lung diseases other than silicosis, as well as those with severe or complex systemic conditions that could confound the analysis such as malignant tumors, severe arrhythmias, heart failure, abnormal thyroid function, blood disease, recent surgery, trauma, long-term alcohol abuse, and poisoning. All experiments were approved by the ethics committee at West China Fourth Hospital of Sichuan University (HXSY-EC-2023025). The need for written patient informed consent was waived as this was a retrospective study, which did not affect the welfare and rights of the patients.

### Variables

The study outcomes are the diagnosis of silicosis and the classification of the disease into stages 1–3. The predictor variables are:

**1. Complete blood count testing** such as: basophil count (Bas#, cells/μL), basophil ratio (Bas%, %), eosinophil count (Eos#, cells/μL), eosinophil ratio (Eos%, %), hematocrit (HCT, %), hemoglobin (HGB, g/dL), immature granulocyte count (IMG#, cells/μL), immature granulocyte ratio (IMG%, %), lymphocyte count (Lym#, cells/μL), lymphocyte ratio (Lym%, %), mean cell hemoglobin (MCH, pg), mean cell hemoglobin concentration (MCHC, g/dL), mean corpuscular volume (MCV, fL), monocyte count (Mon#, cells/μL), monocyte ratio (Mon%, %), mean platelet volume (MPV, fL), neutrophil count (Neu#, cells/μL), neutrophil ratio (Neu%, %), neutrophil-to-lymphocyte ratio (NLR, ratio), platelet large cell count (P-LCC, 10^3^/μL), platelet larger cell ratio (P-LCR, %), platelet crit (PCT, %), platelet distribution width (PDW, %), platelets (PLT, 10^3^/μL), red blood cell count (RBC, 10^6^/μL), red blood cell distribution width coefficient of variation (RDW-CV, %), red blood cell distribution width standard deviation (RDW-SD, fL), white blood cell count (WBC, cells/μL). The complete blood counts were performed by the BC-6800PLUS Analysis System (Mindray, China). PLCC, P-LCR, RDW-CV, and RDW-SD were presented as ratios.
**2. Biochemical testing** such as: albumin/globulin ratio (A/G, ratio), albumin (ALB, g/dL), alanine transaminase (ALT, U/L), aspartate transaminase (AST, U/L), blood urea nitrogen (BUN, mg/dL), cholesterol (CHOL, mg/dL), creatine kinase (CK, U/L), creatine kinase muscle and brain isoenzyme (CKMB, U/L), creatinine (CREA, mg/dL), C-reactive protein (CRP, mg/L), direct bilirubin (DBIL, mg/dL), gamma-glutamyl transferase (GGT, U/L), glucose (GLU, mg/dL), hydroxybutyrate dehydrogenase (HBDH, U/L), high-density lipoprotein-cholesterol (HDL-C, mg/dL), lactate dehydrogenase (LDH, U/L), low-density lipoprotein-cholesterol (LDL-C, mg/dL), total bilirubin (TBIL, mg/dL), triglyceride (TG, mg/dL), total protein (TP, g/dL), and uric acid (UA, mg/dL). The biochemical analysis was performed by Atellica CH930 (Siemens, Germany).
**3. Coagulation testing** such as: activated partial thromboplastin time (APTT, s), D-dimer (DD, μg/mL), fibrinogen (FIB, g/L), prothrombin time-international normalized ratio (PT-INR, ratio), prothrombin time (PT, s), thrombin time (TT, s). The coagulation analysis was performed by a CS-5100 analyzer (Sysmex, Japan).

### Study size

The sample size was estimated using G^*^Power version 3.1.9.2. The minimum sample size required for power analysis (b-error 95%; effect size 0.5) was 105 samples. An equal number of cases and controls were randomly selected. The control sample was retained if there was no statistically significant difference in age and gender compared to the silicosis. Subsequently, subjects were randomly divided into training and testing cohorts in a 2:1 ratio. Training and testing samples were retained if there was no statistically significant difference in age ranges.

### Statistical analysis

Data processing and statistical analysis were conducted using Excel (Version 2016) and R programming (R Foundation for Statistical Computing, Version 4.2). Three machine learning algorithms Random Forest (RF), Least Absolute Shrinkage and Selection Operator (LASSO) regression, and Support Vector Machine-Recursive Feature Elimination (SVM-RFE) were employed for biomarker selection. RF models, implemented using the randomForest package, consist of multiple decision trees constructed from random subsets of predictors. LASSO regression, executed with the glmnet package, is an extension of generalized linear regression that reduces model complexity by penalizing the loglikelihood function. SVM-RFE is a supervised learning method used for classification and feature selection with the e1071 package. To enhance robustness, biomarkers common to all three methods were selected if statistically different in case and control groups. For categorical variables, comparisons between groups were evaluated through the chi-square test. For continuous variables, we used the *t*-test in case of normally distributed variables and the Mann-Whitney test otherwise. Differences between the two groups in selected biomarkers were further evaluated through the heatmap, the Venn diagrams and line plots generated using the ggplot2 package. Finally, confounding variables were excluded from the potential set of selected predictors and the multicollinearity was studied. The remaining biomarkers were included in a logistic regression model implemented in R using the glm function, refined through stepwise regression (using the step function) and multicollinearity analysis. The receiver operating characteristic (ROC) curves were generated using the pROC package to assess the diagnostic performance of the selected biomarkers individually and in combination. All statistical analyses were two-tailed, and results were considered statistically significant if the *P*-value was < 0.05. The diagnostic accuracy of the selected model was further evaluated across the different stages (1–3) of the disease.

## Results

### Participants

We analyzed 306 silicosis cases and 306 controls. Of the 306 patients with a diagnosis of silicosis, 75 had stage 1 silicosis, 65 had stage 2 silicosis, and 166 had stage 3 silicosis. The training cohort included 204 silicosis patients and 204 controls, while the testing cohort included 102 silicosis patients and 102 controls.

### Descriptive data

The mean age of the silicosis group and control group was 55.54 and 55.15 years, respectively. Specifically, since high-intensity physical industries such as coal mining and stone tool making have a clear gender preference, the gender of the silicosis group and the control group in this study was male. There was no statistically significant difference in age and gender between the silicosis group and the control group (*P* > 0.05). Moreover, the mean age of the case and control groups in the training dataset was 55.22 and 55.02 years old, respectively. The mean age of the case and control groups in the test data set was 56.18 and 55.39 years old, respectively. There were no statistically significant differences between the case and control groups in the training dataset and the test dataset in terms of age range or gender (*P* > 0.05, Supplementary Table S1).

### Blood cell parameters, biochemical and coagulation characteristics

As shown in Table 1, the silicosis patients had significantly lower levels of Bas#, Bas%, Eos%, HCT, HGB, Lym#, Lym%, MCH, MCV, MPV, P-LCC, P-LCR, PDW, RBC, and WBC (*P* < 0.05). Meanwhile, significantly higher levels of IMG#, IMG%, Mon#, Mon%, Neu#, Neu%, PLT, NLR, and RDW-CV were observed in silicosis patients (*P* < 0.05). However, the blood cell parameters Eos#, MCHC, PCT, and RDW-SD showed no significant differences between the two groups (*P* > 0.05).

**Table 1:** Comparison of whole blood cell parameters between the silicosis group and the control group.

**Parameters**	**Control group (204)**	**Silicosis group (204)**	***t*-values**	***P*-values**
Bas#	0.03 ± 0.02	0.02 ± 0.01	−2.63	< 0.050
Bas%	0.51 ± 0.28	0.40 ± 0.22	−5.37	< 0.001
Eos#	0.17 ± 0.15	0.15 ± 0.12	−1.83	0.068
Eos%	2.89 ± 2.25	2.46 ± 2.01	−2.52	< 0.050
HCT	47.88 ± 3.62	42.91 ± 5.08	−13.95	< 0.001
HGB	155.4 ± 12.12	139.18 ± 17.94	−13.11	< 0.001
IMG#	0.01 ± 0.02	0.04 ± 0.12	4.39	< 0.001
IMG%	0.22 ± 0.31	0.43 ± 0.84	4.26	< 0.001
Lym#	1.71 ± 0.56	1.30 ± 0.48	−9.54	< 0.001
Lym%	29.02 ± 7.51	20.72 ± 8.02	−13.21	< 0.001
MCH	30.77 ± 1.80	30.14 ± 2.63	−3.48	< 0.001
MCHC	324.56 ± 7.38	324.01 ± 10.72	−0.74	0.457
MCV	94.79 ± 5.08	92.92 ± 6.61	−3.93	< 0.001
Mon#	0.36 ± 0.12	0.46 ± 0.19	8.43	< 0.001
Mon%	6.00 ± 1.47	6.99 ± 1.98	6.95	< 0.001
MPV	11.53 ± 1.59	10.86 ± 1.76	−4.95	< 0.001
Neu#	3.72 ± 1.27	4.87 ± 2.38	7.45	< 0.001
Neu%	61.57 ± 7.99	69.43 ± 9.14	11.33	< 0.001
NLR	2.37 ± 1.07	4.61 ± 4.04	9.38	< 0.001
P-LCC	63.46 ± 16.33	57.3 ± 16.63	−4.62	< 0.001
P-LCR	36.6 ± 10.81	32.12 ± 11.74	−4.91	< 0.001
PCT	0.21 ± 0.05	0.2 ± 0.06	−0.47	0.64
PDW	16.48 ± 0.33	16.34 ± 0.38	−4.82	< 0.001
PLT	183.79 ± 55.23	195.21 ± 71.27	2.21	< 0.050
RBC	5.06 ± 0.43	2.91 ± 0.97	−35.59	< 0.001
RDW-CV	13.32 ± 0.76	13.63 ± 1.35	3.5	< 0.001
RDW-SD	44.74 ± 2.64	44.72 ± 3.76	−0.07	0.946
WBC	5.99 ± 1.65	3.19 ± 2.52	−16.2	< 0.001

Among the biochemical parameters, there were no significant differences in ALT, BUN, DBIL, and GGT levels between the two groups (*P* > 0.05). However, other biochemical parameters were altered to varying degrees. Specifically, the levels of A/G, ALB, CHOL, CK, CREA, GLU, HDL-C, LDL-C, TBIL, UA, TG, and TP were significantly lower in the silicosis group, and the levels of AST, CKMB, HBDH, LDH and CRP were significantly higher in the silicosis group (*P* < 0.05, Table 2).

**Table 2:** Comparison of biochemical parameters between the silicosis group and the control group.

**Parameters**	**Control group (204)**	**Silicosis group (204)**	***t*-values**	***P*-values**
A/G	1.99 ± 0.33	1.55 ± 0.32	−16.63	< 0.001
ALB	48.06 ± 2.74	39.94 ± 4.13	−28.64	< 0.001
ALT	26.4 ± 25.86	27.92 ± 49.57	0.47	0.635
AST	24.19 ± 11.08	31.73 ± 63.32	2.05	< 0.050
BUN	5.65 ± 1.49	5.64 ± 1.78	−0.05	0.958
CHOL	4.89 ± 0.93	4.26 ± 0.83	−8.93	< 0.001
CK	131.44 ± 299.12	88.38 ± 48.47	−2.49	< 0.050
CKMB	15.16 ± 4.84	20.51 ± 6.32	11.77	< 0.001
CREA	82.43 ± 15.97	75.21 ± 32.79	−3.46	< 0.001
CRP	1.48 ± 2.01	19.87 ± 23.37	13.71	< 0.001
DBIL	5.18 ± 1.89	4.96 ± 5.77	−0.65	0.516
GGT	40.35 ± 29.89	45.58 ± 49.09	1.59	0.111
GLU	5.88 ± 1.44	2.71 ± 1.61	−25.67	< 0.001
HBDH	130.22 ± 21.69	157.78 ± 34.76	11.77	< 0.001
HDL-C	1.32 ± 0.34	1.10 ± 0.31	−8.42	< 0.001
LDH	174.66 ± 30.26	233.88 ± 166.76	6.11	< 0.001
LDL-C	3.19 ± 0.83	3.01 ± 0.87	−2.74	< 0.050
TBIL	14.52 ± 6.19	13.34 ± 8.14	−2.02	< 0.050
TG	1.91 ± 1.38	1.40 ± 0.61	−5.86	< 0.001
TP	72.69 ± 4.28	66.68 ± 5.86	−14.48	< 0.001
UA	385.84 ± 83.04	348.7 ± 93.2	−5.21	< 0.001

Compared with the control group, coagulation parameters of PT, PT-INR, DD, FIB, and APTT from the silicosis patients were increased (*P* < 0.05), while there was no significant difference in the level of TT between the two groups (Table 3).

**Table 3:** Comparison of coagulation parameters between the silicosis group and the control group.

**Parameters**	**Control group (204)**	**Silicosis group (204)**	***t*-values**	***P*-values**
APTT	30.44 ± 4.98	31.48 ± 4.33	2.77	< 0.050
DD	94.95 ± 80.31	341.83 ± 425.36	9.98	< 0.001
FIB	2.78 ± 0.51	3.54 ± 0.92	12.74	< 0.001
PT-INR	0.99 ± 0.21	1.06 ± 0.25	3.67	< 0.001
PT	10.99 ± 2.29	11.8 ± 2.68	4.02	< 0.001
TT	14.91 ± 1.65	14.69 ± 1.68	−1.58	0.113

### Machine learning for screening silicosis biomarkers

**Figure 1:**
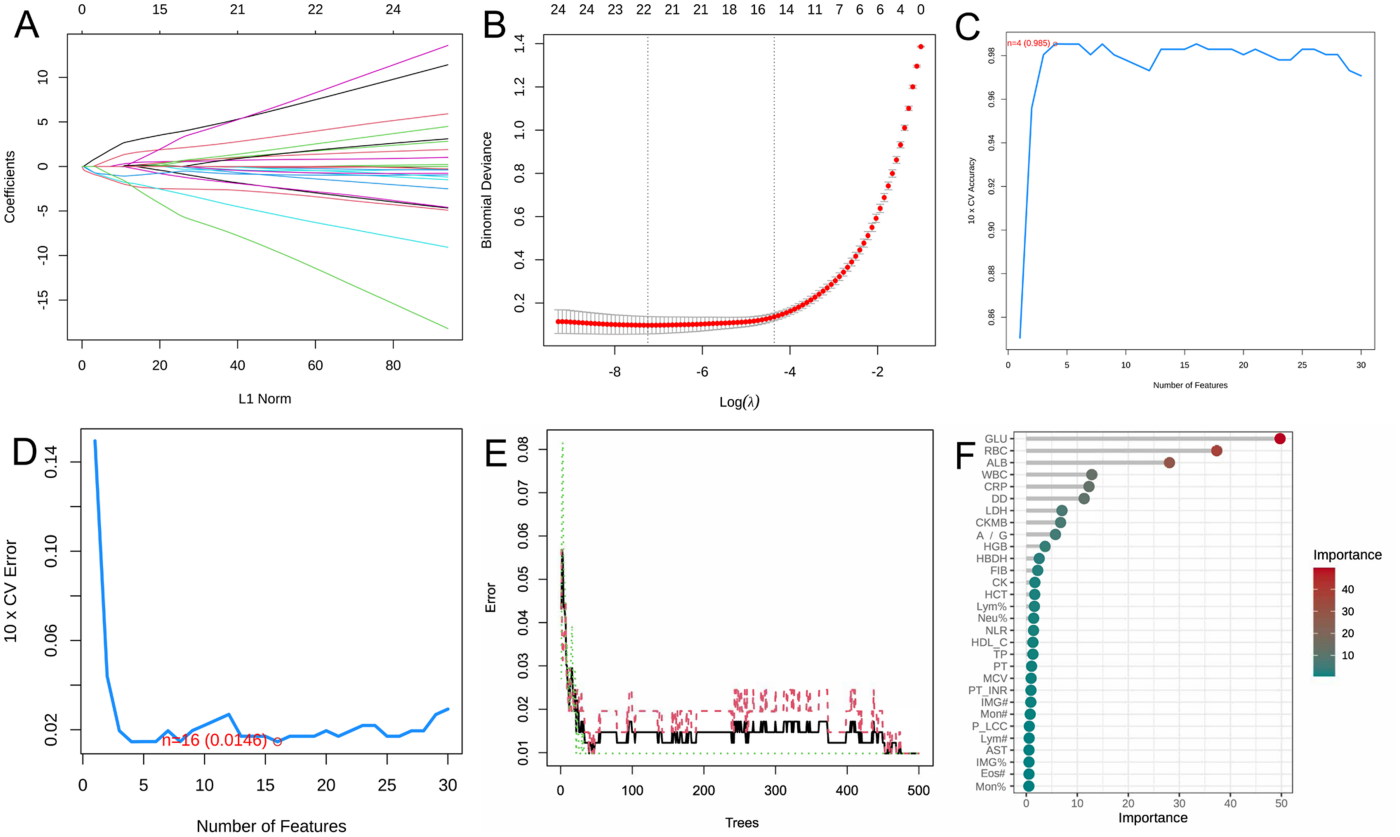
Screening of potential biomarkers with machine learning algorithms. **(A)** LASSO coefficient profiles of the variables. A vertical line was drawn at the value chosen by 10-fold cross-validation. As the value of λ decreased, the degree of model compression increased and the function of the model to select important variables increased. **(B)** The cross-validation results. The value in the middle of the two dotted lines is the range of the positive and negative standard deviations of log(λ). The dotted line on the left indicates the value of the harmonic parameter log(λ) when the error of the model is minimized. Nineteen variables were selected when log(λ) = −7.02. **(C)** Twenty genes were selected based on the SVM-RFE algorithm with the highest accuracy. **(D)** Twenty genes were selected based on the SVM-RFE algorithm with the lowest error accuracy. **(E)** The error rate of random survival forest. **(F)** The top 30 genes were selected and ranked based on the RF algorithm's importance score.

The LASSO regression, SVM-RFE, and RF yielded 18 (Figures 1A, B), 14 (Figures 1C, D), and 13 (Figures 1E, F) biomarkers, respectively. To improve the robustness of the biomarker set, we crossed the biomarkers screened by the three machine learning algorithms, LASSO, SVM, and RF, and finally developed a biomarker set that included eight potential diagnostic biomarkers for silicosis (A/G, ALB, CRP, DD, GLU, LDH, RBC, and WBC).

**Figure 2:**
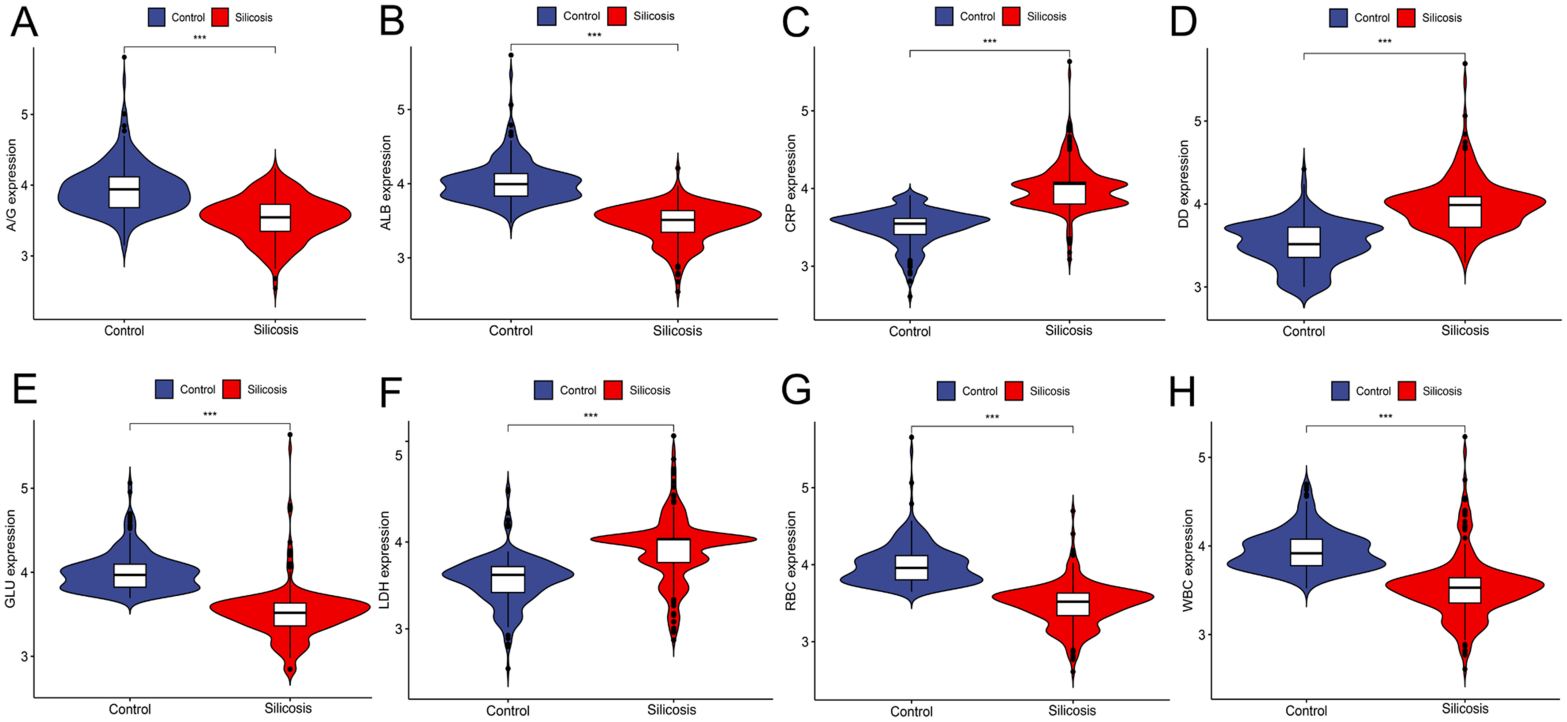
Violin plots of eight biomarker levels from two groups in the training cohort. **(A)** A/G; **(B)** ALB; **(C)** CRP; **(D)** DD; **(E)** GLU; **(F)** LDH; **(G)** RBC; **(H)** WBC. *P < 0.05; **P < 0.01; ***P < 0.001; ****P < 0.0001.

Violin diagrams were further applied to visualize eight potential silicosis biomarkers (Figures 2A–H). Among selected biomarkers, WBC, RBC, ALB, GLU, and A/G showed lower levels in the silicosis group, while LDH, DD and CRP had the opposite trend.

### Construction of logistic regression model based on selected biomarkers

**Figure 3:**
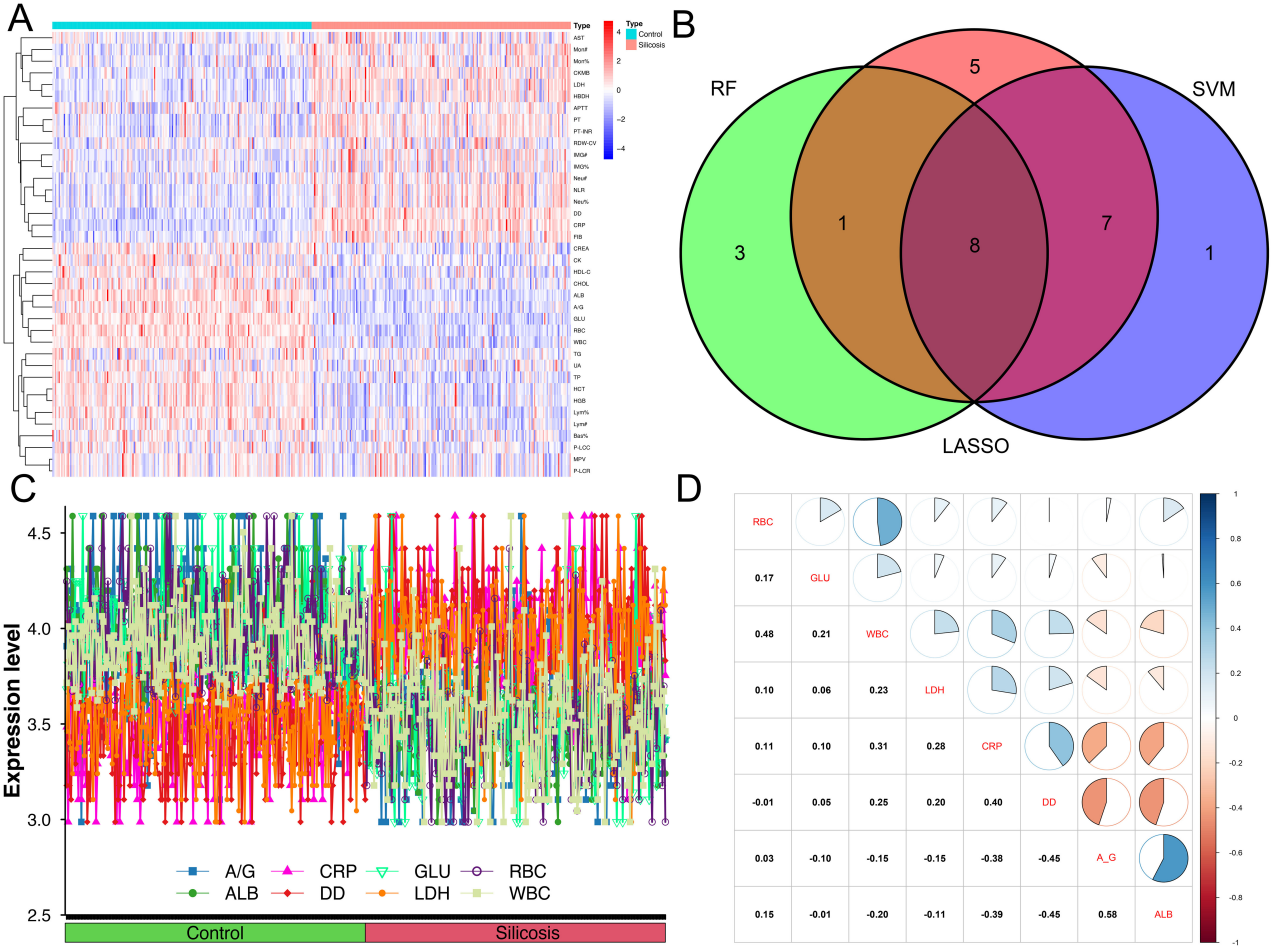
Difference indicators between the two groups and the correlation between eight intersecting biomarkers. **(A)** Heatmap of indicators that differentiate between silicosis patients and controls; **(B)** Venn diagrams of three machine algorithms; **(C)** line graph of eight intersecting biomarkers between silicosis patients and controls; **(D)** the correlation between eight intersecting biomarkers.

The heatmap (Figure 3A) showed the distribution of differential parameters between the silicosis group and the control group. The Venn diagram (Figure 3B) showed the intersection of screening results of three machine learning algorithms. Line diagrams (Figure 3C) showed the differences between the two groups for eight potential key biomarkers. Each of these graphs provided valuable information for assessing the diagnostic potential of these biomarkers. Subsequently, a multi-parameter logistic regression model was developed based on the biomarker set. Among eight selected parameters, GLU and ALB are usually influenced by the short/long-term dietary pattern, and CRP is often increased by stochastic factors such as upper respiratory tract infections. Thus, GLU, ALB and CRP were eliminated from the set. The model was further optimized by eliminating non-significant parameters (RBC) through stepwise regression and eliminating parameters with multicollinearity problems. Given the multicollinearity of strongly correlated independent variables in the logistic model, this study analyzed the correlation between eight key biomarkers, and no strong correlation was observed (Figure 3D). A model composed of A/G, DD, LDH and WBC that independently influenced silicosis diagnosis was established by the logistic regression (*P* < 0.001, Table 4). The logistic regression model is represented as logit *P/(1*–*P)* = −1.985 – 3.113^*^A/G + 7.162^*^DD + 3.393^*^LDH – 6.841^*^WBC. By inputting the predictive indicators into the model, the predicted value (probability P) can be obtained. Encouragingly, the model demonstrated a diagnostic accuracy of 0.931.

**Table 4:** . Construction of a logistics regression model for the diagnosis of silicosis by multiple biomarkers.

**Parameters**	**β-values**	**Wald-values**	**OR (95% CI)**	***P*-values**
Intercept	−1.985	0.102	–	< 0.001
A/G	−3.113	12.124	0.044 (0.007–0.232)	< 0.001
DD	7.162	38.091	1,289.736 (160.029–15,530.358)	< 0.001
LDH	3.393	19.861	29.762 (7.276–146.968)	< 0.001
WBC	−6.841	52.803	0.001 (0–0.006)	< 0.001

“–” indicates that there is no data here.

OR,. odd ratio; CI, confidence interval.

### Independent evaluation of the proposed multi-biomarker logistic model

**Figure 4:**
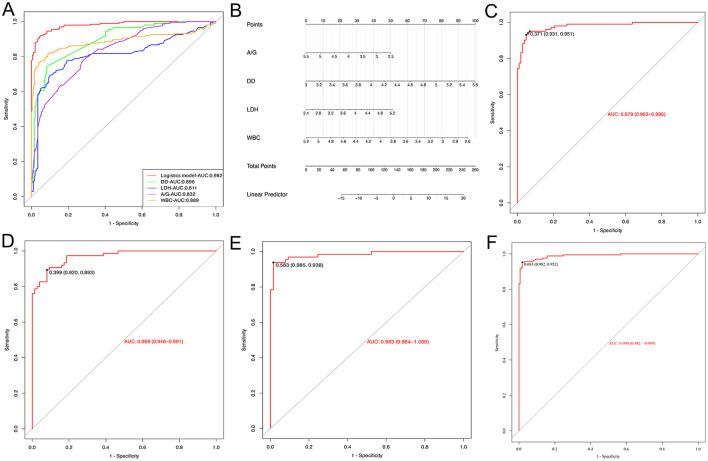
Evaluation of the logistic model. **(A)** ROC curves for the logistic model used to predict silicosis vs. single laboratory biomarkers. **(B)** The Nomogram of the logistic model. **(C)** The ROC curve for the logistic model in the evaluation set. **(D)** ROC curve of the logistic model for stage 1 silicosis. **(E)** ROC curve of the logistic model for stage 2 silicosis. **(F)** ROC curve of the logistic model for stage 3 silicosis.

To assess the predictive value of the proposed multiple biomarker-based logistic model for silicosis diagnostics, the AUC of the logistic model was compared with each of the separate biomarkers. The ROC curve showed that the logistic model had an AUC of 0.982, which was better than that of any single biomarker (Figure 4A). By utilizing the maximum Youden index as a criterion, the cut-off values, AUC, sensitivity, and specificity for each biomarker and the logistic model were determined (Table 5). The logistic model exhibited a sensitivity of 94.6% and a specificity of 95.6% at a threshold value of 0.500. The predictive nomogram was constructed, whereby each biomarker's relative level corresponded to a score, and the total score was obtained by the summation of the score of each biomarker (Figure 4B). Next, the performance of this model was tested in the independent test cohort, obtaining a predictive accuracy of 0.936 and an AUC of 0.979 (Figure 4C).

**Table 5:** A/G, DD, LDH, and WBC diagnostic value for silicosis.

**Biomarkers**	**AUC (95% CI)**	**Cut-off values**	**Sensitivity**	**Specificity**	***P*-values**
A/G	0.832 (0.794–0.87)	3.762	0.721	0.779	< 0.001
DD	0.896 (0.867–0.926)	3.772	0.917	0.745	< 0.001
LDH	0.811 (0.765–0.856)	3.807	0.902	0.691	< 0.001
WBC	0.889 (0.852–0.925)	3.707	0.902	0.824	< 0.001
Logistic model	0.982 (0.972–0.992)	0.553	0.954	0.922	< 0.001

To explore the diagnostic performance of the logistic model in different stages of silicosis, the diagnostic accuracy and AUC of the model at stages 1, 2, and 3 of silicosis were compared. The results showed that the diagnostic accuracy of the logistic model for stage 1, stage 2, and stage 3 silicosis was 88.0, 95.4, and 94.3%, respectively, with AUC of 0.968, 0.983, and 0.990 (Figures 4D–F).

## Discussion

Silicosis is a progressive pneumoconiosis characterized by interstitial fibrosis following exposure to silica dust. According to the Guidelines for the use of the International Labor Organization (ILO) International Classification of Radiographs of Pneumoconiosis, the diagnosis of silicosis primarily includes X-ray posteroanterior chest radiographs and a clear history of silica dust exposure (26). Although silicosis patients may exhibit varying degrees of respiratory symptoms and signs, as well as abnormalities in certain laboratory tests, none of these are specific. In recent years, with the continuous deepening of research on silicosis, some valuable silicosis diagnostic biomarkers have been proposed, such as interleukin-6 (IL6), interleukin-8 (IL8), TNF-α, Clara cell secretory protein 16 (CC16), nephronectin (NPNT), and serum HO-1, shedding light on the pathogenesis and progression of silicosis (19, 27, 28). Furthermore, studies have found that KL-6, surfactant protein D (SP-D) and MMPs are potential biomarkers for diagnosing and monitoring the progression of various fibrotic lung diseases (29). These findings suggest the necessity of applying biomarkers to detect silicosis, in addition to imaging studies and a clear occupational exposure history. However, these biomarkers rely on specialized equipment and expensive consumables, making it difficult to adapt to the widespread regional and population distribution of silicosis. For example, IL-6 and TNF-α are measured by enzyme-linked immunosorbent assays (ELISAs), while KL-6 is measured by electrochemiluminescent immunoassays (ECLIAs), which are expensive and time-consuming. Therefore, we screened routine physical examination indicators with shorter turn-around time (TAT), lower test costs and wider popularity for potential biomarkers related to the progression of silicosis cases. In addition, laboratory indicators have been integrated using mathematical statistical techniques, which is more beneficial than focusing on a single indicator or system to comprehensively describe the pathological process of the disease and improve the sensitivity and specificity of diagnosis.

Previous studies have shown that inhaled silica particles (< 10 μm) reach the distal lung chambers via the mucociliary defenses and may interact with mononuclear alveolar macrophages to induce silicosis (30). Alveolar macrophages either leave the lung when exposed to silica or migrate to the lung interstitium where they are transformed into activated interstitial macrophages. They play a critical role in the progression of silica-induced lung lesions. Our study found that monocyte-related parameters, such as Mon#, Mon%, Neu#, and Neu%, were significantly increased in the silicosis group (*P* < 0.05), while Lym# and Lym% were significantly decreased, ultimately leading to a reduction in the WBC count. Macrophages originate from monocytes entering the lung from the bloodstream, and alveolar macrophage activation leads to changes within the mononuclear cell system. The increased NLR in the silicosis group is attributed to increased neutrophils and decreased lymphocytes in silicosis patients. Serum ALB and globulin are generally used to indicate nutritional status and chronic inflammation, respectively. Recently, the A/G ratio has emerged as a novel prognostic factor (31). In this study, a lower A/G ratio was observed in the silicosis group, which may be due to both lower ALB levels in the silicosis group and an increase in inflammation-related globulin. Inflammation-associated pulmonary fibrosis has been reported in many studies on the pathogenesis of silicosis, confirming that silicosis is closely associated with inflammatory factors. Clinical biochemistry in the silicosis group confirmed significant increases in inflammatory markers including AST, CKMB, CRP, HBDH, and LDH. In particular, the elevation of LDH in patients with silicosis has been confirmed previously, and evaluations of agate and cowboy sandblasting workers have shown that plasma LDH levels are associated with exposure to silica and the severity of the disease (17). Several other conditions, including systemic infection or inflammation, muscle injury, hemolysis, thromboembolism or malignancy, may also result in elevated plasma LDH concentrations. Finally, in the assessment of coagulation function in silicosis patients, we found that APTT, DD, FIB, PT-INR, and PT were higher in silicosis patients compared with the control group, indicating a coagulation dysfunction in silicosis patients. The coagulation and fibrinolysis system maintains a balance under normal conditions, which may be disturbed by hypoxia. A study by Sabit et al. in 2010 showed that 2 h of hypoxic stimulation led to coagulation activation (32). As a product of the degradation of fibrin, the elevation of DD is indicative of hyperfibrinolysis and a hypercoagulable state (33). We speculate that as silicosis progresses, the increasingly severe pulmonary interstitial fibrosis leads to tissue hypoxia and intravascular microthrombosis, which activates the coagulation system and increases DD. This is consistent with the results of Song et al. in pneumoconiosis (34).

The advent of machine learning has facilitated the integration of artificial intelligence with medical data analysis. Through the utilization of its powerful autonomous learning capabilities, machine learning is capable of constructing complex, multivariate predictive models involving multiple parameters. To enhance the efficacy of machine learning in screening, three distinct machine learning algorithms (LASSO, SVM, and RF) were employed to analyze the training set data and to derive their cross-tabulation results. As a new attempt in the study of diagnostic biomarkers for silicosis, this study identified four conventional indicators (A/G, DD, LDH, and WBC) most clinically associated with silicosis, which constitute a validated model for the prediction of silicosis.

Previous research has proposed a few potential biomarkers for silicosis. However, the diagnostic efficacy of a single biomarker is limited when investigating the complex etiology and long incubation period of occupational diseases (13, 35, 36). Therefore, the combination of several biomarkers is necessary to improve diagnostic efficiency. For example, the combined use of KL-6, SP-D and matrix metalloproteinase-2 (MMP-2) for the diagnosis of asbestos and silicosis achieved a sensitivity of 83% and a specificity of 62% (17). In our study, a statistical synthesis approach based on mathematical statistics was proved to provide better analytical results in the investigation of occupational silicosis. The ROC curve analysis showed that the sensitivity of the combination of the four biomarkers was higher than that of any of the single blood biomarkers. The combination of A/G, DD, LDH and WBC can improve the diagnostic efficacy for silicosis. In addition, due to its simplicity, non-radioactive, and non-invasive nature, this model is expected to be widely used for silicosis diagnostics. However, it should be noted that the application of any diagnostic index for silicosis should be carried out in a population exposed to silica dust.

Several limitations of this pilot study should be also acknowledged. Firstly, given the constraints of retrospective data, we were unable to continuously monitor the biomarker levels of each patient to evaluate the dynamic changes in the progression of silicosis. Secondly, further validation through a large-scale, prospective, multicenter cohort study is expected in the future. Additionally, since lung imaging alterations are critical for the current diagnosis of silicosis, future research might integrate patient imaging characteristics to enhance diagnostic precision.

## Conclusions

Based on machine learning, eight potential silicosis biomarkers were selected from routine blood parameters in the clinic, and four biomarker-based (A/G, DD, LDH and WBC) logistic regression model was further developed for the silicosis diagnosis in this study. These routine blood indicators may be used as readily available biomarkers for the adjuvant diagnosis of silicosis. Our study provided a potentially economical, convenient, and efficient strategy for early diagnosis and monitoring of occupational silicosis in occupational disease clinics and primary healthcare institutions.

## Data Availability

The raw data supporting the conclusions of this article will be made available by the authors, without undue reservation.
