# Supplementary material for: Development of a multi-laboratory integrated predictive model for silicosis utilizing machine learning: a retrospective case-control study
Source: Front Public Health. 2025 Jan 15;12:1450439. doi: 10.3389/fpubh.2024.1450439 (PMC11774866; doi:10.3389/fpubh.2024.1450439)
Supplement: Supplementary file 1 [file Table_1.docx]

**Supplementary tables**

**Table S1. Comparison of age and gender between the training and test group.**

| Variable | Training group (204) | | | Test group (102) | | |
| --- | --- | --- | --- | --- | --- | --- |
|  | case | control | p | case | control | p |
| Age (mean) | 55.22 | 55.02 | > 0.050 | 56.18 | 55.39 | > 0.050 |
| Gender (Male/Female) | 204/0 | 204/0 | > 0.050 | 102/0 | 102/0 | > 0.050 |
